# Edible plant extracellular vesicles: An emerging tool for bioactives delivery

**DOI:** 10.3389/fimmu.2022.1028418

**Published:** 2022-12-08

**Authors:** Shi-Jie Fan, Jia-Ying Chen, Chao-Hua Tang, Qing-Yu Zhao, Jun-Min Zhang, Yu-Chang Qin

**Affiliations:** ^1^ State Key Laboratory of Animal Nutrition, Institute of Animal Sciences of Chinese Academy of Agricultural Sciences, Beijing, China; ^2^ Scientific Observing and Experiment Station of Animal Genetic Resources and Nutrition in North China of Ministry of Agriculture and Rural Affairs, Institute of Animal Sciences of Chinese Academy of Agricultural Sciences, Beijing, China

**Keywords:** edible plants, bioactives carriers, anti-inflammation, therapy, extracellular vesicles

## Abstract

The extracellular vesicles (EVs) in edible food have a typical saucer-like structure and are nanoparticles released by numerous cells. They have different components and interact with other biological samples in diverse ways. Therefore, these nanoparticles could be used to develop bioactives delivery nanoplatforms and anti-inflammatory treatments to meet the stringent demands of current clinical challenges. This review aims to summarize current researches into EVs from edible plants, particularly those that can protect siRNAs or facilitate drug transportation. We will discuss their isolation, characterization and functions, their regulatory effects under various physiological and pathological conditions, and their immune regulation, anti-tumor, regeneration, and anti-inflammatory effects. We also review advances in their potential application as bioactives carriers, and medicinal and edible plants that change their EVs compositions during disease to achieve a therapy propose. It is expected that future research on plant-derived EVs will considerably expand their application.

## Introduction

Plant foods, such as grains, vegetables, and fruits, are universal nutritional components of the human diet that can provide proteins, fat, carbohydrates, and vitamins, etc. (1, 2). Plant foods are also the main source of dietary fiber, which potentially benefits human health by reducing laxation and cardiovascular disease (3). There is evidence that plant-based diets are the most protective of our health. Therefore, it is essential to focus on positive food associations and the beneficial constituents of plant foods (4). The protective properties of plant-based diets may be linked to many dietary components, including vitamins, minerals, phytochemicals, and fiber (5). Recent studies have found that plant also contain EVs, which play an important role in various medical fields, such as anti-cancer, anti-inflammation, and bioactives delivery (6, 7).

**Figure 1:**
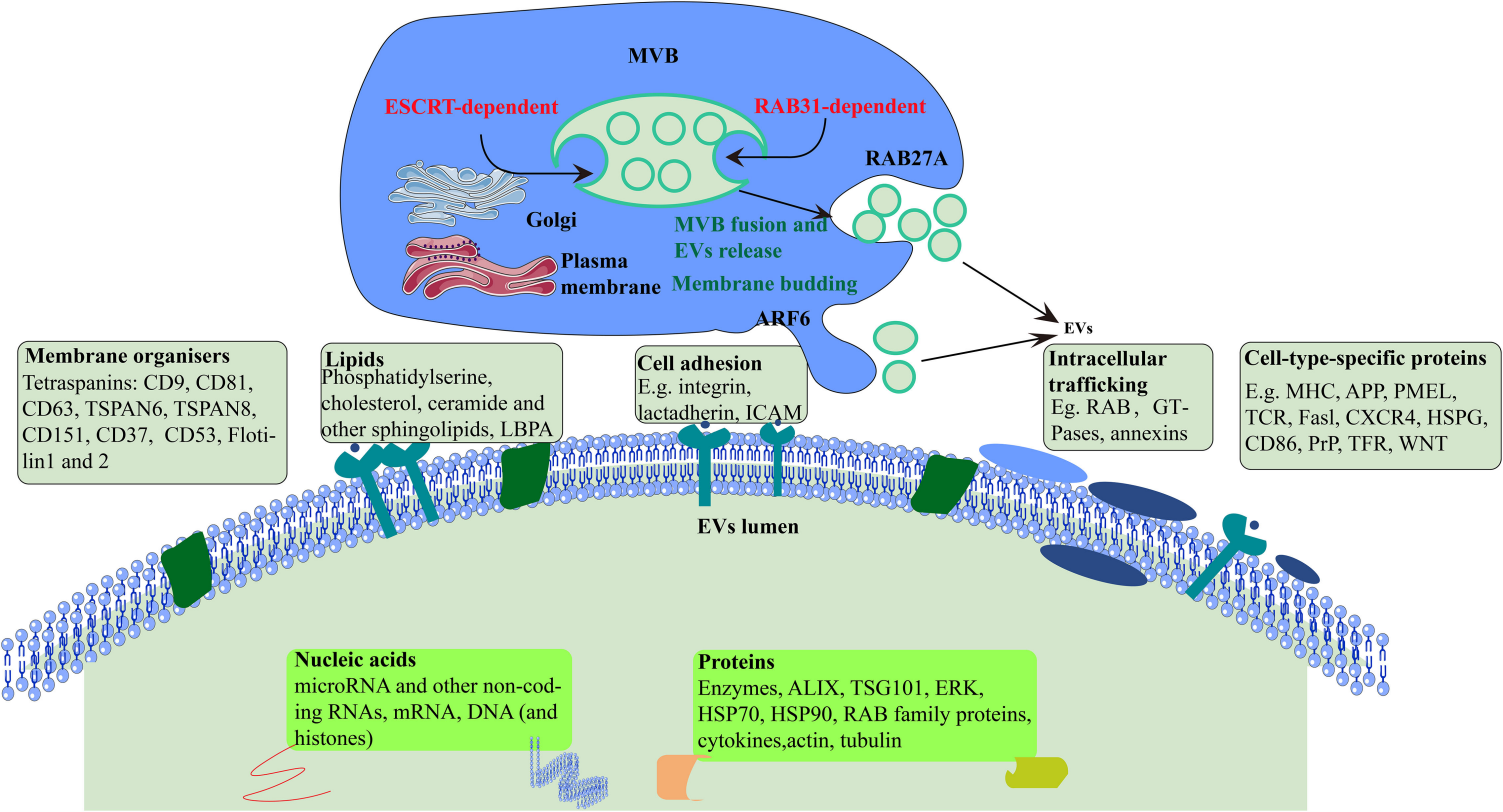
Formation and secretion of EVs. Cellular formation of EVs occurs either ESCRT-dependent or RAB31-dependent pathway. ARF6, ADP-ribosylation factor 6; TSPAN, tetraspanin; LBPA, lyso-bis-phosphatidyl acid; ICAM, intercellular adhesion molecule; MHC, major histocompatibility; APP, amyloid precursor protein; TCR, T cell receptor; CXCR4, CXC-chemokine receptor 4; HSPG, heparan sulfate proteoglycan; PrP, prion protein; TFR, transferrin receptor; TSG101, tumor susceptibility gene 101 protein; HSP70, heat shock 70 kDa protein; HSP90, heat shock 90 kDa protein.

In general, EVs are small vesicles with a membrane structure that are actively secreted by cells. They are mainly divided into three categories, which are exosomes, microvesicles, and apoptotic bodies according to their size, biological characteristics, and formation process (8, 9). They were originally isolated from the red blood cell supernatant of sheep (10) and contained large amounts of proteins (membrane proteins and intracellular proteins), lipids, DNA, and RNA (microRNA, mRNA and other non-coding RNA) (11) that can be absorbed by most cells through endocytosis. Recent studies have shown the potential of exosomes as delivery vesicles for bioactive compounds that may either be part of their cargo, or lipid structure, see **Figure 1**. They have a number of roles in cells, such as promoting cell proliferation, activation angiogenesis, anti-cancer functions, and information delivery between cells. It has also been reported that proteins are involved in EV biogenesis mechanisms (12). EVs in body fluid samples can be identified from their cells of origin, such as those derived from tumor cells, which means that EVs could potentially act as diagnostic biomarkers for human diseases (13–15).

Many studies have suggested that EVs in fruits vegetables, medicinal and edible plants (16, 17) are rich in an heterogeneous array of compounds that have biological activities, such as lipids, proteins, noncoding RNAs, and microRNAs (8). EVs are a particularly important class of vesicle-like substances because they protect labile cargos against degradation and provide a vehicle for cargo uptake through the endocytosis of EVs by almost all tissues (18, 19). In this review, we report on plant EVs biogenesis, biological activities, and bioactive delivery. We highlight the key open questions and technical challenges that currently limit the development of plant EVs. However, we are optimistic that these challenges will be solved in the near future and provide a theoretical basis for the application of edible plant EVs.

## EVs formation and secretion

Earlier studies showed that EVs are synthesized from endosomes in three steps. First, plasma membrane forms endocytic vesicles that fuse to form early endosomes. Next, the early endosome encapsulates material in the body, forming multiple intraluminal vesicles, which further transform into multivesicular bodies. Finally, the multivesicular bodies combine with the plasma membrane and release intraluminal vesicles to the outside of the cell, which are EVs (20).

EVs biogenesis begins with the invagination and budding of the endosomal membrane and then intraluminal vesicles (ILVs) form within a maturing endosome, now called a multivesicular body (MVB) (21). In detail, the mechanism underlying the formation of MVBs and ILVs is mainly driven by the endosomal sorting complex required for transport (ESCRT), apoptosis gene 2-interacting protein X (ALIX), and tetraspanins (CD63, CD81, CD9). Many proteins, such as cytoskeleton proteins and Ras-associated binding protein (RAB), are involved in the cellular transport of EVs. Finally, EVs bind to protruding proteins *via* the soluble N-ethylmaleimide-sensitive fusion protein attachment protein receptor (SNARE) protein complex and are excreted (22). However, it is evident that although ESCRT has always been considered necessary for ILV formation, ILVs in multivesicular endosomes (MVEs) can still be observed in cells lacking ESCRT, indicating the existence of ESCRT-independent ILV formation in cells. RAB31 has dual functions in the biogenesis of EVs. It drives ILVs formation and suppresses MVE degradation, which is an ESCRT-independent EVs pathway(**Figure 1**) (23).

## The landscape of EVs in edible plants

EVs provide preliminary information communication in plants, which promotes plant growth and development, improves defense responses, and elevate symbiosis between plants and microorganisms (24). Plant EVs contain many active ingredients that can improve the body’s immune response. Human beings are omnivorous animals that consume many edible plants as an essential part of their diet. Plant derived EVs can be divided into two categories, one is the vesicular material isolated from plants, and the other is the exogenous addition of plant extracts. The change of EVs released by affecting the body or cells is another type of EVs. Plant EVs are in contact with the intestinal tract throughout our lives. They participate in intestinal tissue renewal processes and have important biological functions against inflammatory diseases (e.g.; colitis injury, liver steatosis) and cancers associated with specific lipid and miRNA contents. Recently, edible plants have gradually become a rich source of EVs, and many EVs from edible plant have attracted considerable attention (**Table 1**).

**Table 1:** Edible plant-derived EVs.

Variety	Extraction	Identification	References
Grape	sucrose density gradient centrifugation	EM, NTA	(6)
Grapefruit	ultracentrifugation	EM, NTA	(25)
Carrot	ultracentrifugation	BCA, EM, NTA	(26)
Ginger	ultracentrifugation	BCA, EM, NTA	(26)
Citrus limon	ultracentrifugation	EM, NTA	(27)
Wheat	extraction kit	Lowry Assay, SEM, FACS	(28)
Blueberry	ultracentrifugation	BCA, DLS, SEM	(29)
Sinensis	ultracentrifugation	BCA, TEM, DLS	(30)
Coconut	ultracentrifugation	SEM, DLS	(16)
Arabidopsis	ultracentrifugation, ultrafiltration, PEG-based precipitation	TEM, WB	(31)
Broccoli Tomato	ultracentrifugation ultracentrifugation	NTA BCA, SEM, MRPS, DLS	(32) (33)
Strawberry	ultracentrifugation	TEM	(34)
Ginseng	ultracentrifugation	TEM, NTA	(35)
Dendropanax morbifera	Ultrafiltration	BCA, DLS, TEM	(36)
Momordica. charantia	sucrose density gradient centrifugation	BCA, NTA, TEM	(37)

EM, electron microscope; NTA, nanoparticle tracking analysis; BCA, bicinchoninic acid; SEM, scanning electron microscope; FACS, flow cytometric; DLS, dynamic light scattering; TEM, transmission electron microscope; WB, western blotting; MRPS, microfluidic resistive pulse sensing.

## Isolation and characterization of plant EVs

The International Society for EVs has emphasized the urgent need to standardize the methods for EVs isolation and quality assessment. Currently there are a variety of EVs isolation methods, for example, blood-derived exosomes are extracted by ultracentrifugation (38), milk exosomes are isolated through sucrose density gradient centrifugation (39), and polyethylene glycol is used to abstract exosomes from cell culture media (40). As shown in **Table 1**, EVs in edible plants are mostly extracted using ultracentrifugation, where > 100,000×*g* speeds are used (41). However, the purity of the EVs obtained by a single ultracentrifugation method is not high, which may be due to the fact that plants contain a large number of components that are similar to EVs (42). There is a wide variety of plant raw material resources that produce EVs, which means their cost is low and there are no ethical issues involved. The yields of plant-derived EVs extracted *via* multi-step differential centrifugation combined with sucrose density gradient centrifugation are relatively high, which is very conducive to the large-scale mass production of plant-derived EVs. Mu., et al. used a bicinchoninic acid (BCA) assay and found that 100 g of plant raw materials could extract 320-450 mg EVs by the sucrose step gradient and centrifugation method (26). However, there is an urgent need for a suitable method that can extract plant EVs more efficiently.

How to measure EVs purity is one of the most intractable problems in EVs biological research (43). Accurately measuring and assessing plant EVs purity is a critical issue when evaluating edible plant EVs dosages for functional studies, bioactives delivery, and clinical application. There are a number of ways of characterizing plant EVs, but they are generally based on the analysis and identification of their morphology, size, and marker proteins (44–46), among which, morphology and particle size are the two most common indicators. Morphology identification includes using a transmission electron microscope, an atomic force microscope, and/or a cryo-transmission electron microscope, while particle size analysis includes nanoparticle tracking analysis (NTA) and dynamic light scattering (DLS) (16, 47, 48), the NTA and DLS techniques can also measure EVs production (49, 50)(**Table 1**). In biochemical terms, it is essential to measure EVs activity and concentration by ELISA and BCA (51, 52). Currently, among transmembrane proteins, CD63, CD81, CD9, and heat stock protein70 have been identified as possible markers of animal-derived EVs (53, 54). However, few studies have used protein markers to identify plant EVs, such as western blotting or flow cytometry. This is probably due to the wide variety of potential plant species and their complex protein composition. There has also been relatively little preliminary research on protein markers and plant EVs antibodies are not commercially available. Further studies are clearly needed to identify stable plant EVs markers.

## Edible plant EVs assimilation and utilization

Studies have shown that EVs are very important biological information transmission carriers in the body and that they can participate in the regulation of various physiological processes by mediating through direct cell–cell contact or transfer of secreted molecules (55). However, there have been few studies on how EVs enter and are exploited in the organism. Previous studies have used membrane dyes to mark and observe EVs based on the structure and characteristics of their double-layer membrane (56). EVs have been labeled with green fluorescent protein, then co-cultivation with cells that take them up through endocytosis (57). Cells appear to take up EVs by a variety of endocytic pathways, including clathrin-dependent endocytosis and clathrin-independent pathways, such as caveolin-mediated uptake, macropinocytosis, phagocytosis, and lipid raft-mediated internalization (58). Similar to animal derived EVs, plant EVs can also be labeled with membrane dyes (6).

Researchers use the *in vivo* tracing method to label EVs so that the transportation and distribution of EVs in the body can be tracked (59). Cy7 has been used to mark EVs that fluoresce *in vivo* (60). EVs can influence the function of cells or tissues far from where they are secreted in the body (61). After intravenous injection with Cy7 labeled mesenchymal stem cell-derived EVs, imaging analysis showed that most EVs were present in the liver, kidney, and lung (62). DIR can be stably bind to EVs. DiI fluorochrome was incubated with milk-derived exosomes, adipose-derived stem cell exosomes, and exosome-like nanoparticles from coconut water, can be taken up by bacteria (63). DIR-labeled ginger exosomes-like nanoparticles (G-ELNs) were injected into mice, and the mouse intestine and liver were imaged and analyzed. Ginger-derived EVs were observed to transfer from the intestine to the liver (17). This is conducive to reveal the absorption mechanism of plant-derived EVs in the body and improve the utilization efficiency.

## Biology of EVs in edible plants

EVs play an important role in carrying proteins, RNA, lipids, and they can be released by most cells, including normal and cancer cells. Current studies mainly focus on animal-derived EVs, which play important roles in many physiological and pathological processes, but the biology of plant-derived EVs remained unclear. **Table 2** shows the differences between plant-derived EVs and animal-derived EVs. This information can provide references for studies on plant EVs.

**Table 2:** Comparison of plant-derived EVs and animal-derived EVs.

Type studies	Plant-derived EVs	Animal-derived EVs
Extraction methods	ultracentrifugation and sucrose density gradient centrifugation	ultracentrifugation, sucrose density gradient centrifugation, size-based techniques, precipitation, immunoaffinity capture-based techniques and microfluidics based techniques
Particle size	EVs 100−1000 nm	microvesicles100-1000 nm exosomes 30-150 nm
Lipids	phosphatidylethanolamine, phosphatidic acid, phosphatidylcholine, no cholesterol	cholesterol, sphingomyelin, ceramide
Proteins	less research, contains less than animal-derived EVs, proteins that regulate carbohydrate/lipid metabolism, membrane proteins etc.	rich in variety, mainly targeting fusion proteins, rab family proteins, heat shock proteins family, transmembrane proteins, cytoskeleton proteins etc.
RNA	mainly miRNA and small amount of ribosomal RNA	mRNA, miRNA, lncRNA, cirRNA and lack of ribosomal RNA
Application	anti-tumor, anti-inflammation, and drug delivery	anti-tumor, anti-inflammation, and biomarkers of diseases
Advantages	abundance of plant resources, large-scale production from abundance of plant resources, high biocompatibility and bioavailability with low toxicity, suitable features for a drug delivery system	high homology, can be used as a biomarker of disease, diversity of separation methods
limitations	concern about poor biocompatibility from impurities, fewer targeting moieties for mammalian cells	low production, less resources

The Extracellular RNA Communication Consortium (ERCC) aims to promote the development of extracellular RNA (exRNA) biology and to identify whether exRNAs and their carriers, including EVs, mediate intercellular communication and have clinical applications (64). A systematic comparison among 10 exRNA isolation methods for five biofluids revealed significant differences in the complexity and reproducibility of the small RNA sequence profiles. An interactive web-based application (miRDaR) has been developed to help researchers select the optimal exRNA isolation method (65). RNA-seq data from cerebrospinal fluid, serum, saliva, plasma, and urine samples, and 5309 exRNA-seq samples were collected from 19 different studies to develop an atlas that can provide a basis for exRNA researches. Further analysis of the ex-miRNA-seq of 462 samples from 17 different diseases found that biological samples from different sources contained specific miRNAs. Then, a website focusing on ex-miRNA expression profiles was developed, which will aid researches into exosome biomarkers, miRNA functions, liquid biopsies, and clinical applications (66).

miRNAs are important small non-coding RNAs that play important roles in the post-transcriptional regulation of biological processes. The expression profiles of miRNAs vary widely in normal and disease tissues and they exhibit condition-specific characteristics (67). The EVs derived from most cell lines contain mRNAs and miRNAs (68) that can be absorbed by cells *via* endocytosis (69). Other studies have revealed that different miRNAs have different functions (70). For example, they can be used as biomarkers, in gene medicines, and for intercellular communication. Studies have shown that Alzheimer (AD) patients derived serum EVs had a large number of AD-related miRNAs compared to volunteers. Therefore, exosomal miRNAs have great application prospects in the pathophysiological process, diagnostic biomarkers and treatment of AD (71). Many stem cell-derived exosomal miRNAs can be used to treat various diseases, such as cancer (72, 73), senescence (74), cardiovascular disease (75), and promote bone regeneration (76). Large numbers of miRNAs in plant-derived EVs have been detected (26). Xiao et al. analyzed the miRNA profiles of EVs and identified a total of 418 miRNAs in fruits and vegetables. There were 32 to 127 per species in 11 different fruits and vegetables. They found commonalities and differences in miRNA species and expression levels in different plants (77). Researchers have also found differences in miRNA content after undertaking a miRNA-seq analysis with real-time PCR verification of immature and mature coconut water EVs. A total of 47 known miRNAs were found, most of which were highly expressed in mature coconut water EVs (16). Ginger-derived EVs contain 125 different miRNAs, of which 124 miRNAs have been predicted to have putative human targets (78). Zhang et al. reported that plant-derived miR-168a could penetrate the mammalian gastrointestinal (GI) tract and enter the liver (79). However, the physiological relevance of such cross-kingdom regulation has been debated due to the decreased stability of plant-derived miRNAs during the cooking process and GI digestion. An alternative route by which such cross-kingdom regulation can be achieved is *via* miRNAs present within EVs. Kalarikkal and Sundaram plotted the relative abundance of the SARS-CoV-2 targeting miRNAs as a heat map in 11 edible plant derived nanoparticles (ENPs). They identified 22 miRNAs that could potentially target SARS-CoV-2 genome. Eleven miRNAs showed absolute target specificity towards SARS-CoV-2 (80). Studies on exosomal miRNA have become more prevalent because it plays an important role in various fields. Plants are abundant sources of EVs whose miRNAs are similar to the human genome. Therefore, a large number of plant EVs miRNAs may provide a theoretical basis for the treatment of diseases.

Proteomic studies of EVs released by primary cell cultures, cell lines, tissue cultures, or isolation from biofluids have provided extensive catalogues of protein abundance in different types of EVs (38, 81). Théry’s team carried out an extensive study to isolate different populations of small EVs. According to this team, Exosome has four transmembrane proteins (CD9, CD63, CD81), syntenin-1, TSG101 and ADAM10 (82). In addition, exosome is highly rich in cholesterol, sphingomyelin and hexosylceramide at the expense of phosphatidylcholine and phosphatidylethanolamine (83). EVs also contained the amyloid precursor protein, prion protein and DJ-1, biomarkers for neurodegenerative diseases. Hence, the authors postulated that α-synuclein (α-syn) and Aβ peptides could be involved in the dissemination of the disease to other parts of the brain (84). The vesicle surface information, known as EVs characteristics, can be used to target the brain with nanoparticles or free drugs (85). Plant EVs also contain abundant proteins, compared to animal-derived EVs, whereas there is less protein in plant-derived EVs (**Table 2**). Studies have found that ginger-derived nanoparticles (GDNPs) had a low proteins, which tended to be predominantly cytosolic, such as actin and proteolysis enzymes. There were also few membrane proteins, such as membrane channel/transporters (e.g., aquaporin and chloride channels) (78). A total of 598 proteins have been identified in *Arabidopsis* that are enriched when they act as stress-response proteins (86). Furthermore, the tomato root EVs proteome contains proteins involved in the oxidative stress response, such as annexin p34, calmodulin, and patatin-like protein 2, which are reported to regulate the response against *Botrytis cinerea* infection, resistance against *Phytophthora infestans*, general plant immunity, and calcium-mediated signal transduction (33).

Generally, EVs are enriched in cholesterol and sphingolipids, indicating that their membrane composition resembles that of lipid rafts (87, 88). Lipids are also abundant in plant EVs. The components of lipids in EVs are vary from different plants and include phosphatidyl ethanolamine (PE), phosphatidylcholine (PC), phosphatidic acid (PA), digalactosyl-diacylglycerol (DGDG), monogalactosyldiacylglycerol (MGDG), phosphatidylinositol (PI), and phosphatidylserine (PS) (25, 78, 89–91). Lipidomic data indicate that ginger-derived EVs are enriched with PA (53.2%) and PE (26.1%) (6). The data also indicates that both GDN and GDEN2 is enriched with PA (37.03 and 40.41%, respectively), digalactosyldiacylglycerol (39.93 and 32.88%, respectively), and monogalactosyl monoacylglycerol (16.92 and 19.65%, respectively) (90).

The body adjusts EVs contents in response to external disturbances, such as alternation of cold and heat, hypoxia, and oxidative stress, resulting in changing to EVs proteins and RNAs (92–94). Plants show species differences and regional characteristics, which means that the composition and content of EVs from different plant sources vary. Studies have shown that the miRNA contained in grape-derived EVs was mainly miR119 and that its RNA content was much lower than that of ginger-derived EVs (6, 26). However, ginger-derived EVs also contain lower amounts of proteins (78). How to classify EVs from different plants will become an important future research topic.

## Plant EVs functions: A world of possibilities

EVs are nano-scale vesicles that are actively secreted by most cells, which make them important in cell-to-cell communication and various diseases (95). In the field of animal EVs, EVs that are biomarkers for clinical diagnosis, and circulating exosomes and their encapsulated miRNAs correlated well with atherosclerosis severity, suggesting that they could have potential diagnostic properties (96). Furthermore, exosomes in nerve cells change with the different stages of neurodegenerative disease, which means that they could potentially act as disease biomarkers (97, 98). Some researchers also labeled the lipid bilayer of EVs by using a biotin labeled 1,2-distearoyl-sn-glycerol-3-phosphoethanolamine polyethylene glycol (DSPE-PEG) probe. Labeled EVs in plasma can then be collected through magnetic submicron particles (MMPs) coated with NeutrAvidin (NA) (99). Wang et al. found a silica based liposome nanoprobe that can enrich EVs in pancreatic cancer plasma, and EVs can be used to monitor pancreatic cancer (100). In addition, endogenous exosomes are capable of effectively increasing the concentration of therapeutic circulating exosomes around the infarct area, which is important after myocardial infarction (MI). This suggests that the biodistribution of endogenous exosomes can be regulated to improve cardiac functional restoration after MI (101). Mesenchymal Stem Cell-Derived EVs can effectively reduce mitochondrial damage and inflammation in animal models of cell and kidney damage by increasing mitochondrial transcription factor A expression, mtDNA damage in target cells and leakage of cytosolic mtDNA (62). They may also promote angiogenesis, bone regeneration, skin regenerative repair, etc. In other researches, bacterial extracellular vesicles (BEVs) are also widely, Pathogenic bacteria use the BEVs as conduit for transferring virulence factors, including enzymes, DNA, and small RNAs to their host cells, leading to cell damage and inflammatory responses. Besides, BEVs are produced as decoys to neutralize the host’s immune system reactions against the invading bacteria (102, 103).

Studies on the EVs from edible plants are developing rapidly. They have highlighted the prospects for EVs in edible plants and their functions. Edible plant-derived EVs have broad application as cancer, anti-inflammatory, and antiallergic treatments. Bioactives delivery is currently an important research field in plant EVs, including RNA, anti-cancer drugs, etc. The current application of plant EVs to various diseases mainly includes the following aspects.

Host *Arabidopsis* cells secrete exosome-like EVs that can deliver small RNA to the fungal pathogen *Botrytis cinerea*, silence pathogenic genes, and increase antibacterial properties (31). Proteomic analyses have shown that *Arabidopsis*-derived cell vesicles were highly enriched in proteins involved in biotic and abiotic stress responses. This finding suggested that EVs might make a significant contribution to plant immune responses (86). Wheat-derived EVs can promote the proliferation and migration of human dermal fibroblasts, human umbilical vein endothelial cells, and human keratinocyte cells, increase the expression of the wound healing gene *COL1A*, stimulate fibroblasts, promote angiogenesis, and accelerate skin wound healing (28). Garlic derived external vesicles can alleviate liver injury and dietary obesity by inhibiting NLRP3 inflammatory corpuscles (104). Other studies have indicated that grapefruit nanocarriers (GNVs) can deliver chemotherapy drugs, siRNA, DNA expression vectors, and proteins to different types of cells. The target specificity of GNVs *in vivo* has been demonstrated by co-injecting therapeutic bioactives with folic acid, because they significantly improved the efficiency of cells that expressed targeted folate receptors. In two tumor animal models, the inhibition of tumor growth by enhanced chemotherapy further confirmed the therapeutic potential of GNVs (25). Teng et al. reported that exosome-like nanoparticles (ELNs) from edible plants such as ginger were preferentially taken up by gut bacteria in an ELN lipid-dependent manner. The ELN RNAs notably enhance gut barrier function to alleviate colitis by regulating gut microbiota composition and host physiology (17). The study found that the isolated nanovesicles inhibited the proliferation of cancer cells in different tumor cell lines by activating TRAIL-mediated cell apoptotic *in vitro (* 27). Grape ELNs can mitigate dextran sulfate sodium (DSS) -induced colitis by inducing lgr 5 stem cells and then rapidly initializing tissue renewal (**Table 3**) (6). Grape-derived EVs can upregulate heme oxygenase 1 and reduce interleukin (IL)-1β and tumor necrosis factor-α (TNF-α) levels. They can also act as immunomodulators to maintain the homeostasis of intestinal macrophages and reduce DSS-induced colitis in mice (89). Furthermore, edible plant derived ELN treatment of mice leads to Wnt-mediated activation of *Tcf4* transcription in the crypts according to an analysis of intestines from canonical Wnt reporter mice (26). Both nuclear factor erythroid 2-related factor 2 (Nrf2) and Wnt/Tcf4 activation play a critical role in anti-inflammatory responses. Shiitake mushroom-derived ELNs could potentially be used to curb fulminant hepatic failure by suppressing the NLRP3 inflammasome. Their actions include suppressing the secretion of IL-6 and both the protein and mRNA levels of the *Il1b*, Casp1 autocleavage, and pyroptotic cell death. They can also protect mice from D-galactosamine or Lipopolysaccharide (LPS) induced acute liver injury (106). Momordica. charantia-derived EVs-like nanovesicles increased ratios of *p*-AKT/AKT and *p*-ERK/ERK in MCELNs treated irradiated H9C2 cells, and mitigated myocardial injury and fibrosis in a thoracic radiation mice model (37). Strawberry-derived ELNs improve a body’s antioxidant capacity and relieve oxidative stress by enhancing vitamin C activity (34). Zu et al. isolated and identified three types of exosome-like nanotherapeutics (NTs) from small-sized, middle-sized, and large-sized tea leaves. Tea leaf-derived NTs restored intestinal epithelial barrier integrity and upregulated the tight junction protein expression of zonula occluded-1 and mucoprotein 2. The NTs can also decrease TNF-α, IL-6, and IL-10 levels, increase IL-10 levels, and improve anti-oxidation, such as reducing malondialdehyde levels and inhibiting colonic myeloperoxidase activity. The NTs constitute a novel, biocompatible, and economically feasible platform for the prevention and treatment of colon diseases (107).

**Table 3:** Overview of the biological functions of EVs from a variety of plant sources.

Plant	Mediators	Functions	References
*Arabidopsis*	TET8, TAS1c-siR483 TAS2-siR453 SYP61	transferred host sRNAs silence fungal virulence genes and suppress fungal pathogenicity plant immune responses	(31) (86)
Grapefruit		delivery FA	(25)
Ginger	PA, miR7267-3p and IA3 caspase1, IL-1β, IL-18 and NLRP3 Nrf2	ease IBD inhibitory effects on activation of the NLRP3 inflammasome protecting liver function	(17) (105) (90)
Citrus Limon	TRAI	inhibit cancer cell proliferation and suppress CML xenograft growth	(27)
Grape	Lgr5+ stem cells	protection against dextran sulfate sodium (DSS)-induced colitis	(6)

TET8, tetraspanin8; SYP61, syntaxin protein61; FA, folic acid; IA3, indole-3-carboxaldehyde; NLRP3, nucleotide binding oligomerization domain like receptors;

TRAI, tumor necrosis factor (TNF)-related apoptosis inducing ligand- receptor, IBD, inflammatory bowel disease.

In particular, we focused on ginseng, which is well-known for its multiple pharmacological properties, including anticancer, anti-obesity, and neuroprotective activities, and is used as a medicinal herb or a dietary supplement worldwide. Extracts from ginseng, such as ginsenoside (unique triterpenoid saponins), phenols, and acidic polysaccharides, have been shown to exhibit numerous pharmacological efficacies. Ginseng-derived nanoparticles significantly suppressed IL-4 and IL-13-induced M2-like polarization of macrophages *via* control of toll-like receptor 4 and myeloid differentiation factor 88, thereby inhibiting tumors (35). The G-ELNs contain lipids, proteins, and RNAs and are easily taken up by macrophages. Treatment with G-ELN suppresses the downstream inflammasome activation pathways, including caspase1 autocleavage, IL-1β and IL-18 secretion, and cell apoptosis (105). The G-ELNs strongly inhibit NLRP3 inflammasome activation, which provides a theoretical basis for inflammation control *via* regulation of NLRP3 inflammasomes in the acute process. Studies have shown that oral GDNPs could promote liver detoxification and anti-oxidation by activating the Nrf2 pathway, thereby protecting liver function in the alcohol liver mouse model induced by alcohol (90). All of these results provide a theoretical basis for the application of plant-derived EVs to diseases (26) and indicate that plant EVs have extensive future prospects.

## Plant EVs as bioactive-delivery nanoplatforms

**Figure 2:**
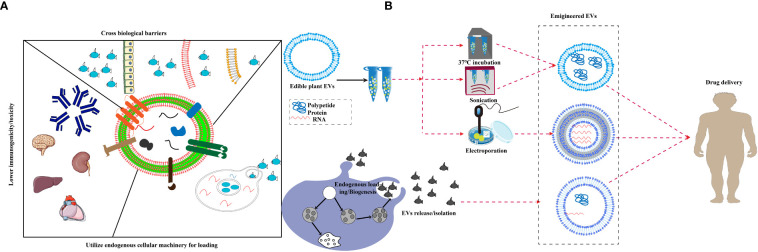
Bioactives delivery methods for edible plant-derived EVs. **(A)** Proposed unique features of extracellular vesicles, EVs are believed to be able to cross various biological barriers and deliver cargo across endosomal membranes, and display lower toxicity in spleen, brain and liver as well as reduced immunogenicity. **(B)** A schematic diagram displaying the strategy for endogenous loading of engineered EVs, EVs package exogenous substances through ultrasound, co-incubation, electroporation and chemical modification, then pass various biological barriers.

So far, about 150 anti-cancer drugs based on nanotechnology have been shown to have favorable applications, some of which have been widely used in clinical studies, such as liposomes and protein nanoparticles (108). An emerging research focus is on EVs that have been broadly applied in delivering anti-cancer, anti-inflammation and other drugs (109). As the most important nerve center, the brain has a special blood brain barrier, which makes it difficult for foreign substances to reach. Therefore, the therapeutic window for brain diseases is extremely narrow (110). In the past decade, exosomes have emerged as novel therapeutic effectors in immune therapy, regenerative medicine and drug delivery. The production of biological products has been terminated due to the occurrence of adverse immune reactions to nano drugs, such as allergic reaction, cytokine release syndrome, neutralization of biological activity, cross reaction with endogenous protein counterparts and non-acute immune reaction (111). In addition, the miRNA of Plant EVs regulate the communication between the intestinal flora and the host immune system, transforming it into a stable balance between the immune system and the intestinal flora (32). EVs is widely claimed to be biocompatible because of its mammalian cell origin and “physiological” composition (112). They are characterized by several favorable features, such as low immunogenicity, biodegradability, low toxicity, encapsulating endogenous bioactive molecules, strong protection for cargo and the ability to cross the blood-brain barrier (BBB). Yang T et al. evaluated the exosome-mediated delivery across BBB barrier (BBB) and explained the transport mechanisms. In this study, zebrafish embryos were injected with rhodamine 123-loaded exosomes *via* the cardinal vein, and fluorescence of rhodamine 123 was examined in the circulating system *in vivo*. When given alone, rhodamine 123 remained within the blood vessels and was not observed in the brain tissue. In contrast, when delivered by brain endothelial bEND.3 cells-derived exosomes, rhodamine 123 showed significant penetration in brain regions, confirming the ability of exosomes to deliver drugs across BBB for brain diseases treatment (113). MC-ELNs, exosome-like nanoparticles from Momordica charantia, have the capacity of crossing the BBB, reducing infarct size, and improving neurological deficits in the cerebral ischemia-reperfusion injury (114). EVs delivery can make up for the side effects of chemotherapy drugs. The latest research shows that EVs delivery can significantly reduce the side effects of the anti-cancer drug doxorubicin on the heart and significantly inhibit the tumor effect (115). Recent studies have shown that intravenous injection of EVs containing siRNA is more effective than injection of lipid nanoparticles containing siRNA in inhibiting pancreatic cancer in mice (56). It is reported that the mice took grapefruit derived EVs and DOTAP-DOPE (1,2-dioleoyl-3-trimethylammonium-propane) liposomes, and the levels of alanine aminotransferase, aspartate aminotransferase and proinflammatory cytokines in serum and tissues of the mice taking liposomes increased significantly, while had no difference in grapefruit derived EVs (25). These results showed that Plant EVs have significant non immunogenicity. Compared with synthetic drug carriers, Plant EVs have a strong supporting immunomodulatory effect, which can achieve tissue homeostasis and contribute to the health of organisms (41). Studies have shown that grapefruit EVs can circulate in the peripheral blood of cancer mice model, which makes it easy for grapefruit EVs to carry drugs into tumor tissues and function (7). A series of transactions by large pharmaceutical companies showed that the industry is embracing EVs to deliver nucleic acid drugs to tissues that are hard to target. In recent years, some new companies have appeared that aim to focus on the development of therapeutic exosomes or other EVs as delivery vehicles for genes and RNA drugs because they are natural nucleic acid vectors (116). A large number of plant species can be used as resources for EVs that have conversion function and can convert substances that are hardly absorbed by human bodies into easily-absorbed ones (89, 117). Compared to synthetic drug carriers, plant-derived EVs have better biocompatibility, lower immunogenicity, and can pass through various physiological barriers more easily, such as the BBB, which leads to safer drug delivery. There are two research ideas about how to load foreign substances into EVs. One is to load them into cells so that the EVs secreted by the cells carry the load and the other is to load exogenous substances directly after separation of EVs using co-incubation, ultrasound, and electroporation techniques (118). Plant EVs possess features that qualify them as a potential drug delivery system, plant EVs are believed to be able to cross various biological barriers, including tissue barriers and plasma membranes, and deliver cargo across endosomal membranes. The established cell system is used to directly ingest drugs and load them onto secreted EVs, such as exosomes derived from curcumin-treated (primed) macrophages where the incorporation of curcumin into exosomes can increase the solubility, stability, and bioavailability of curcumin (119, 120)(**Figure 2A**). Under most conditions, the sonication technique is used to attract oppositely charged drugs, such as doxorubicin, which improves drug loading efficiency (108). Another study also demonstrated that chemotherapeutic drugs and siRNAs could be encapsulated into nanovectors without affecting their biological effects *in vivo (* 25) as shown in **Figure 2B**. Passion fruit-like Exo-PMA/Au-BSA@Ce6 nanovehicles were fabricated using fresh urinary exosomes loaded with multi-functionalized PMA/Au-BSA@Ce6 nanoparticles using an instant electroporation strategy. The Ce6 uptake by Exo-PMA/Au-BSA@Ce6 encapsulation increased about 1.6-fold compared to that of PMA/Au-BSA@Ce6 and free Ce6 (121). Therefore, plant EVs have innate bioactive delivery advantages.

EVs have similar characteristics to secreting cells, reflect the physiological and pathological changes in cells, and can act as diagnostic markers for diseases (122). In recent years, it has been reported that plants and plant extracts play an important role in immunity, tumors, and cardiovascular diseases (123, 124), which has further promoted studies on plant EVs.

### Curcumin

**Figure 3:**
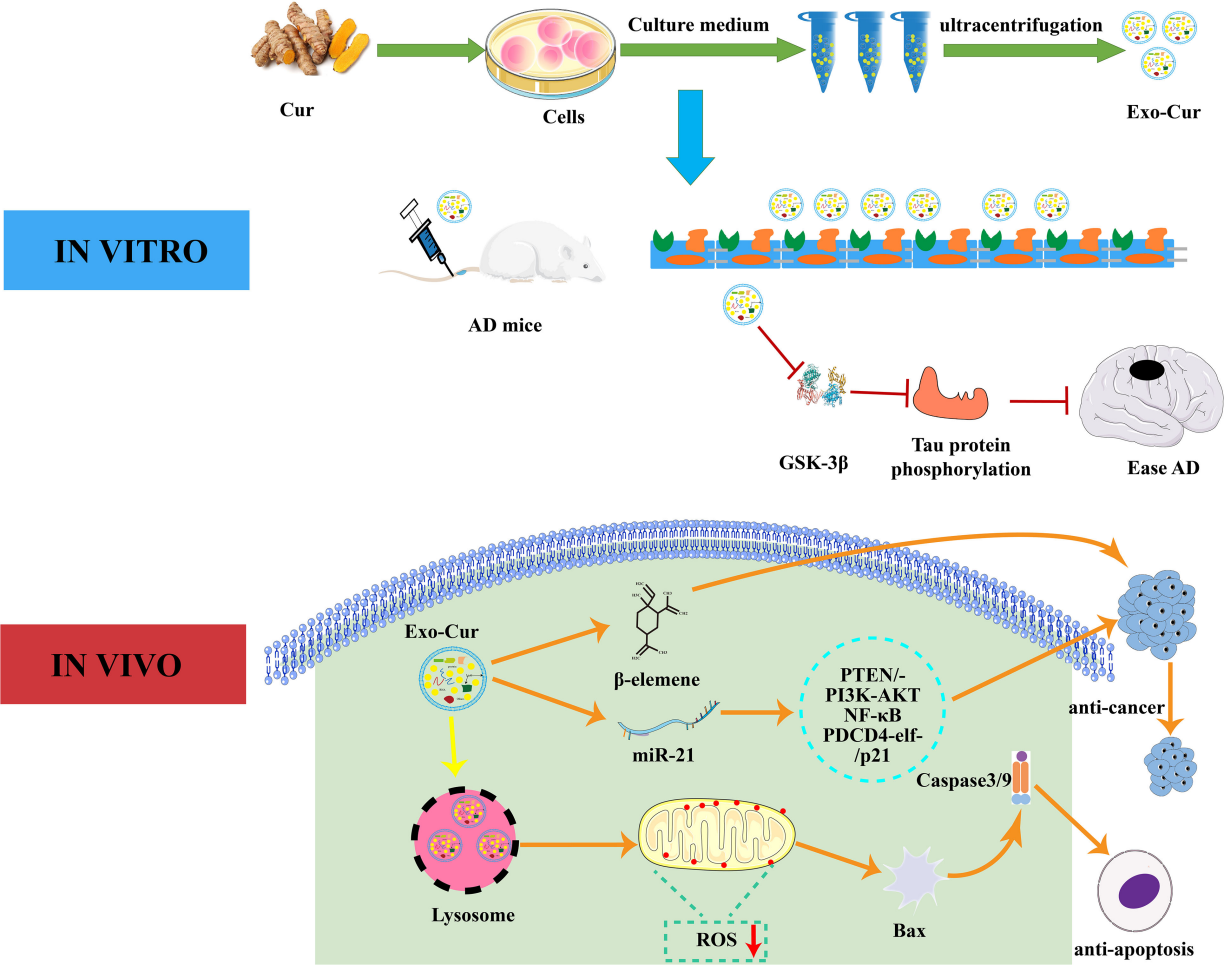
Interaction mechanism between EVs and curcumin. *In vitro*, curcumin-primed exosomes potently ameliorate cognitive function in AD model by inhibiting hyperphosphorylation of the Tau protein through the AKT/GSK-3β pathway. *In vivo*, Exo-Cur mediates various effects on cancer cells including proliferation, apoptosis, metastasis and anti-cancer drug resistance. Cur, curcumin; Exo, Exosomes; AD, Alzheimer’s disease; PTEN/PI3K/AKT, phosphatase and tensin homolog/phosphoinositide 3-kinase/protein kinase B; PDCD4, programmed cell death protein 4; GSK-3β, glucogen synthase kinase-3 beta; BBB, blood brain barrier.

Curcumin is a natural polyphenol extracted from turmeric and is used in Chinese medicine, spices, and as a food colorant (125). It is able to modulate various targets in a variety of cell types due to its well-known anti-autoimmune, anti-cancer, and anti-inflammatory properties (126). Exosomes derived from curcumin-treated cells reduce LPS-mediated inflammation by inhibiting IL-1β, TNF-α, IL-6, and other inflammatory factors (127). Transcription factor 21 (TCF21) is a marker for lung cancer and can be suppressed by methylating transferase DNA (cytosine-5-)-methyltransferase 1. Curcumin inhibits lung cancer by up-regulating TCF21 in EVs (128). Exosomes derived from curcumin-treated (primed) cells alleviate oxidative stress induced by high homocysteine levels through enhancement of tight junction (ZO-1, claudin-5, occludin), and adherent junction (VE-cadherin) proteins, and endothelial cell layer permeability (120). As shown in **Figure 3**, curcumin can effectively cross the BBB after being absorbed by EVs. It can combine cell-derived exosomes by activating the Protein kinase B/Glycogen synthase kinase-3 (AKT/GSK-3β) pathway to inhibit phosphorylation of the tau protein, which prevents neuronal death and relieves AD symptoms *in vivo (* 119). Curcumin has great potential to improve targeted bioactives delivery and restore neuronal function in AD therapy. MiRNA-21 can increase cell proliferation and decrease apoptosis and therefore increase cancer incidence (129). Studies have shown that curcumin reduces miRNA-21 levels by accelerating the efflux of miRNA-21 in exosomes and inhibiting transcription of the miRNA-21 gene. The down-regulation of miRNA-21 inhibits proliferation and the differentiation of tumor cells through its effects on the phosphatase, tensin homolog/phosphoinositide 3-kinase (PI3K)/Akt and the nuclear factor kappa-B (NF-κB) pathway (130). Its downregulation can improve the bioavailability of curcumin and miR-144-3p by combining curcumin with heart-targeted EVs. This process could potentially protect the heart and treat MI (131). The current co-incubation of curcumin and EVs could have significant clinical roles, and curcumin-encapsulated EVs show good therapeutic potential.

### Grapefruit

**Figure 4:**
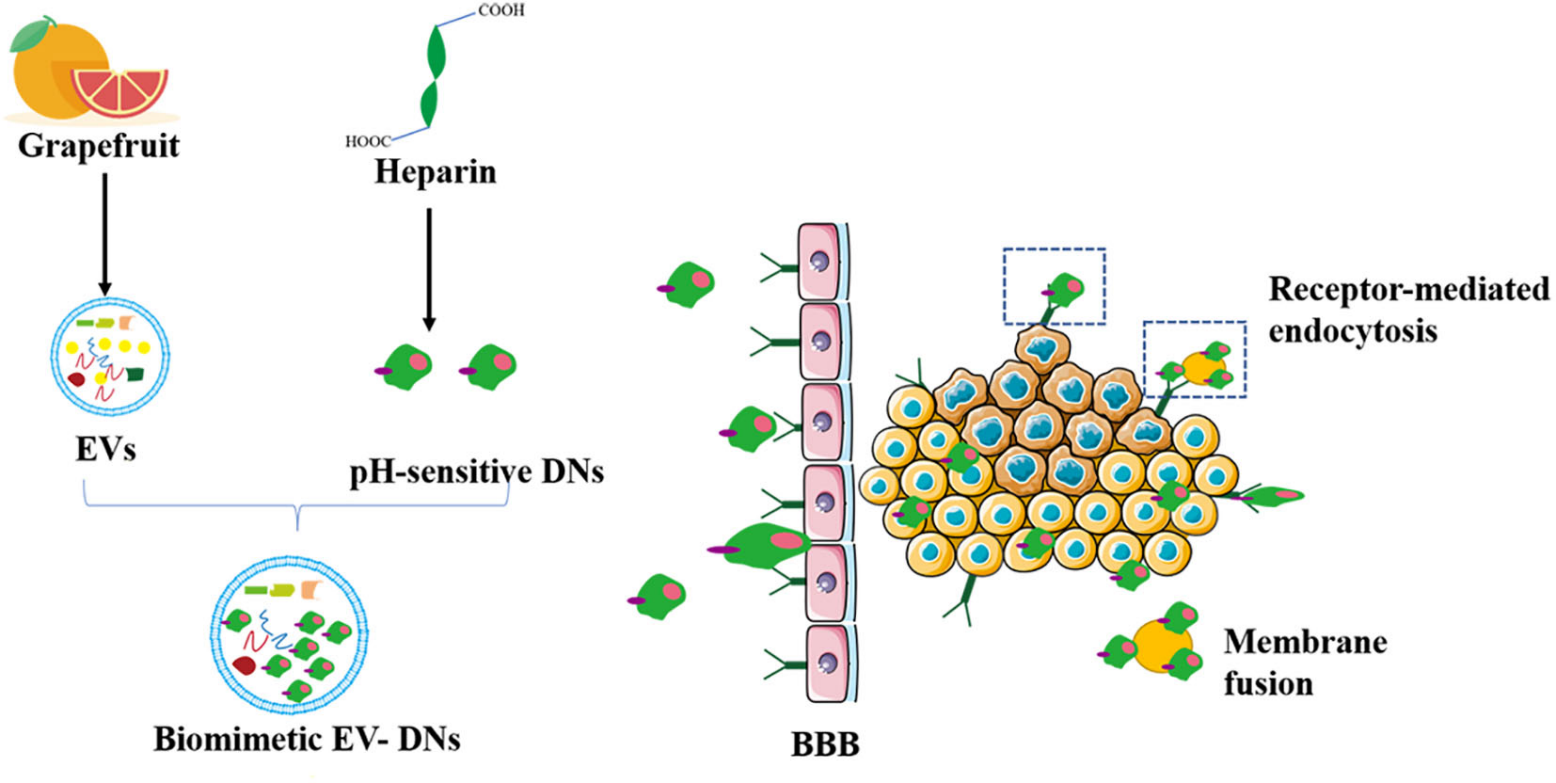
Grapefruit EVs-doxorubicin loaded nanoparticles for glioma treatment. The pH-sensitive DNs as a carrier were devised to incorporate to the surface of grapefruit EVs for the treatment of glioma by natural biomacromolecule heparin. DN, doxorubicin-loaded heparin-based nanoparticles; BBB, blood brain barrier.

Many components in citrus fruits have physiologically active functions. For example, many have antioxidant, anti-cancer, antibacterial, and antiviral effects (132, 133). Importantly, grapefruit EVs can be modified to achieve specific cellular targeting (25). Researchers have packaged miR-18a in grapefruit EVs by targeting *Irf2* and macrophages that synthesize interferon-γ. This leads to the production of IL-12, which activates natural killer cells and inhibits the metastasis of colon cancer cells (134). As shown in **Figure 4**, a study has patched doxorubicin-loaded heparin-based nanoparticles (DNs) onto the surface of natural grapefruit EVs to develop an EVs that is rich in DNs. This EVs can also protect the effective transportation of DNs, thereby significantly improving the efficacy of anti-glioma compounds. EVs increase drug loading by 4 times compared to traditional drug loading systems. They can also pass through the BBB and enter the glioma tissue by binding to the receptor and through exocytosis, which greatly promotes the internalization and anti-proliferation ability of cells, increases the utilization of drugs and maximizes the inhibition of glioma proliferation (60).

### Ginger

Recent studies have shown that ginger has anti-inflammatory and anti-tumor effects (135). Ginger-derived EVs also have anti-inflammatory effect and relieve alcoholic fatty liver (17, 90). The ginger-derived EVs carriers have high drug loading efficiency. Under ultrasonic treatment, ginger is loaded with negatively charged drugs to form nano-drug particles. Arrowtail RNA nanoparticles on ginger-derived EVs that display ligands for siRNA delivery can inhibit tumors when administered intravenously (47). This provides a broad application prospect for the development of anti-cancer drugs.

## Role of EVs in medicinal and edible plant treatments

In recent years, studies on plant extracts across various fields have become more extensive, such as research into their anti-cancer, anti-inflammatory, and anti-diabetes effects (136–140). Researchers use plant extracts to treat various diseases and have revealed that EVs play an important role in the alleviation of disease by plant extracts.

Medicinal and edible homologous plants play a role by changing the EVs components of mammalian cells, which is different from general plant derived EVs, with disease targeting and high heterogeneity. Unlike plant derived EVs, they are limited by the lack of plant texture and extraction methods. They can achieve anti-cancer and drug loading functions by changing the composition and yield of EVs in the body or cells. Berberine significantly reduces the synthesis of fatty acids in HCT116 cells and Hela cells, thereby affecting the formation and secretion of EVs and inhibiting the growth of some tumors (117). Berberine can reduce the transforming growth factor (TGF)-β1 in exosomes released by high glucose induced podocytes injury *via* the TGF-β1-PI3K/AKT pathway (141). TGF-β1-containing exosomes from high glucose-treated glomerular endothelial cells can activate glomerular endothelial cells to promote renal fibrosis (142). Amla extract can increase release of miR-375 in ovarian cancer, reduce tumor production, and prevent rises in exosomes miR-375 levels (143). Black bean extract can enhance exosomes anti-cancer capacities in various types of cells (144), whereas fermented lingonberry juice has anti-invasive and anti-proliferation effects similar to curcumin. Loading curcumin and into Candida EVs improved its poor bioavailability (145). Lingonberry juice can also reinforce tumor cell anti-proliferation *via* co-incubation with milk derived EVs (146). These results support the idea that EVs can be affected by medicinal and edible plants.

## Conclusions and perspectives

Edible plant EVs can transport substances carried by plants, such as proteins, miRNAs, lipids, or exogenous substances, into animal bodies. Therefore, plant EVs can deliver plant derived signal materials and carry useful materials to animal organs. However, whether the material delivery of edible plant EVs is purposeful or arbitrary has not been shown. In addition, researchers have recently discovered that edible plant EVs can act as nanotherapeutics or as delivery mechanisms for proteins, nucleic acids, and other bioactive cargoes across various biological barriers. However, to date, there is very little information about the biosynthesis of edible plant EVs, their endogenous substances, and the biological functions of edible plant EVs. Edible plant EVs can be derived from many different sources. They are considered as natural bioactive carriers, which is going to greatly accelerate the study in edible plant EVs.

Animal-derived EVs also play an important role in diseases. Their synthesis, secretion, and absorption mechanisms are much better known compared to plants. Therefore, further studies on the mechanisms underlying plant EVs are required. Differential ultracentrifugation to extract plant EVs is the main method used in current studies. However, the equipment required for this extraction method is expensive. Therefore, it is important to develop methods similar to those used for animal-derived EVs. At present, the composition of EVs in plants is not completely understood and the functions of the proteins, nucleic acids, lipids, and other substances contained in EVs are still not clear. In addition, plant EVs are highly diverse so there is an urgent need to develop standardization guidelines for plant EVs research that are similar to those for animal-derived EVs.

The expected expansion of EVs in the treatment and prevention of IBD, skin damage, and other related diseases by medicinal and homologous edible plants has bright prospects in the disease treatment field. Plant regionality, species differences, and growth period have obvious effects on the efficacy of plant based medicines, which means that it is extremely important to detect the EVs active ingredients in specific medicinal and homologous edible plants.

Studies on plant extract uses in the field of alternative antibiotics have become more extensive and plant EVs have an obvious anti-inflammatory function. However, current studies on plant EVs are limited to cell and mouse experiments. Furthermore, how to deliver the correct anti-inflammatory dose to animals needs to be addressed. Analyses of the active ingredients in a variety of plants and how to apply them to enhance anti-inflammatory activity should be the focus of research over the next few decades.

**Figure 5:**
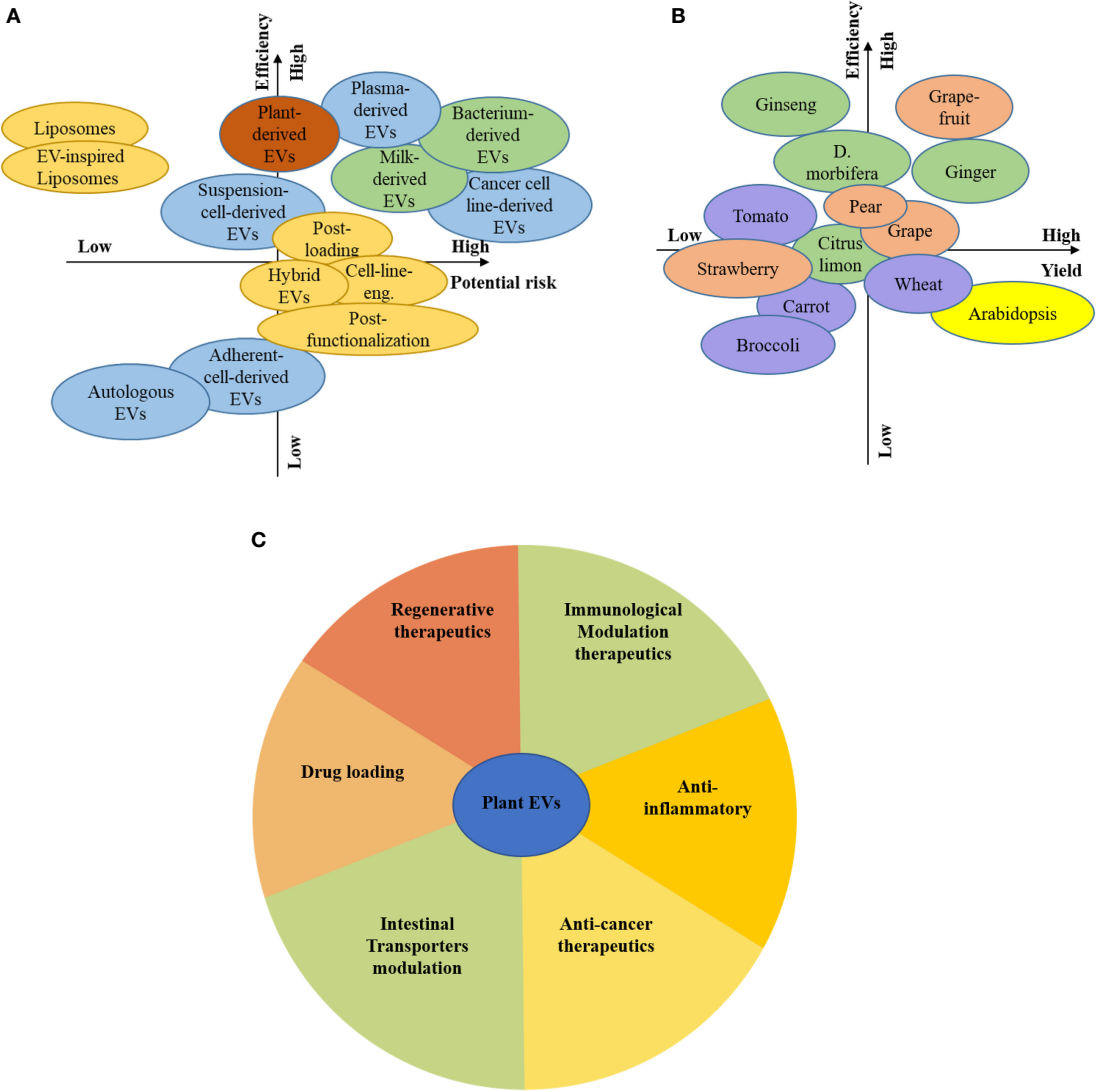
Prospects for plant EVs. Consideration of the different source and associated risks and efficiency **(A)**, Consideration of the plant-based source and associated risks and efficiency **(B)**, The function of plant-derived EVs by colour-coded guidelines **(C)**.

Plant resources are plentiful compared to animals, and cells, can be used as donors for EVs, which means they have broad prospects (**Figure 5A**). There are differences in plant varieties, growth environments, growth cycles, composition, function, and the heterogeneity of plant-derived EVs. The unique medicinal value of edible plants, such as ginger-derived EVs, can affect inflammation, and succulent plants as a source of EVs, such as grapefruit, grape, and broccoli, can be used to deliver siRNA and anti-cancer drugs (**Figures 5B, C**).

Plant EVs are already innately laden with bioactive compounds, and further packaging these nanoparticles with siRNA as bioactives is a challenge. The ultimate target of any drug delivery system is to realize real clinical applications and tangible patient benefits. In order to achieve these goals, there needs to be a robust understanding of how administered plant EVs will be transported *in vivo*, reach the intended tissue target, and deliver their therapeutic cargo. It is accepted that plant EVs can deliver bioactives *in vivo*. However, their conversion efficiency and absorption efficiency are technical challenges. To fully understand the potential of plant EVs-mediated bioactives delivery, we believe that the field must look to intelligently designed strategies that actively exploit the biological characteristics of EVs.

In summary, plant EVs have many advantages, such as low toxicity and strong absorption. They can as nano-therapeutics for diseases (immunological modulation, anti-tumor, treatment of fatty and colitis etc.), and deliver information substances and package bioactive substances so that they can pass through BBB, which can relieve stroke and Alzheimer’s Disease. Over the past few years, research into plant EVs has made great progress, but it also has many challenges. However, these problems will be overcome in the future. Plant EVs are becoming a widespread force in the EV field.

## Author contributions

S-JF reviewed the literature and wrote the manuscript. J-YC Participated in charting. C-HT and Q-YZ contributed to manuscript revision. Y-CQ and J-MZ design the review and revised the manuscript. All authors contributed to the article and approved the submitted version.
